# The Fanconi Anaemia Components UBE2T and FANCM Are Functionally Linked to Nucleotide Excision Repair

**DOI:** 10.1371/journal.pone.0036970

**Published:** 2012-05-15

**Authors:** Ian R. Kelsall, Judith Langenick, Craig MacKay, Ketan J. Patel, Arno F. Alpi

**Affiliations:** 1 Scottish Institute for Cell Signalling, University of Dundee, Dundee, United Kingdom; 2 MRC Laboratory of Molecular Biology, Cambridge, United Kingdom; King Faisal Specialist Hospital & Research Center, Saudi Arabia

## Abstract

The many proteins that function in the Fanconi anaemia (FA) monoubiquitylation pathway initiate replicative DNA crosslink repair. However, it is not clear whether individual FA genes participate in DNA repair pathways other than homologous recombination and translesion bypass. Here we show that avian DT40 cell knockouts of two integral FA genes – *UBE2T* and *FANCM* are unexpectedly sensitive to UV-induced DNA damage. Comprehensive genetic dissection experiments indicate that both of these FA genes collaborate to promote nucleotide excision repair rather than translesion bypass to protect cells form UV genotoxicity. Furthermore, UBE2T deficiency impacts on the efficient removal of the UV-induced photolesion cyclobutane pyrimidine dimer. Therefore, this work reveals that the FA pathway shares two components with nucleotide excision repair, intimating not only crosstalk between the two major repair pathways, but also potentially identifying a UBE2T-mediated ubiquitin-signalling response pathway that contributes to nucleotide excision repair.

## Introduction

Cells are regularly exposed to a large number of genotoxins that would compromise genome integrity. To counteract this threat they activate many DNA damage response pathways [1]. One of these pathways is inactivated in the human genetic illness Fanconi anaemia – a condition that causes developmental abnormalities, bone marrow failure and cancer predisposition [2]. At a cellular level FA patient derived cell lines show a marked hypersensitivity to DNA interstrand cross link (ICL)-inducing agents such as cisplatin and mitomycin C. Exposure to these agents precipitates a high frequency of chromosomal abnormalities. Over the last decade there has been considerable progress in identifying the many genes that are mutated in FA. The analyses of their gene products suggest that most of them work collectively in a common DNA damage response–hitherto referred to as the FA core pathway. A key step in the FA core pathway is the site-specific monoubiquitylation of two FA proteins, FANCD2 and FANCI [3], [4]. These two proteins form a complex (D2/I complex), which accumulates at sites of DNA crosslink damage. The monoubiquitylated D2/I complex is then thought to directly regulate DNA repair by promoting nuclease incision, lesion bypass and processing intermediates of double strand breaks [5], [6], [7], [8], [9].

Terminal protein ubiquitylation usually requires the consecutive action of an enzyme cascade consisting of three classes of enzymes: The E1 ubiquitin activating enzyme, E2 conjugating enzymes and finally E3 ubiquitin ligases. In the FA core pathway the enzymes corresponding to this cascade reside in UBE2T (E2) [10] and the nuclear multisubunit FA core complex (E3) [11]. This core complex consists of most of the cloned FA gene products (FANC-A, -B, -C, -E, -F, -G, -L, and –FM) and also the FA protein associated molecules (FAAP16, FAAP24, FAAP100 and MHF1/2) [2]. At the heart of this core complex resides one central molecule that is regarded as the crucial component of this E3 ligase – the FANCL subunit. This molecule consists of the N-terminal ubiquitin conjugating (UBC)-like domains ELF and DRWD, and the C-terminal RING type zinc finger domain [11], [12], [13]. RING domains are signatures for a large protein family of E3 RING ligases [14]. It is no surprise that whilst the RING domain in FANCL is dispensable for assembling the core complex, it is essential for FANCD2 monoubiquitylation [10], [11], [15]. Furthermore, in a minimal *in vitro* assay FANCL and UBE2T are necessary and sufficient for the site-specific monoubiquitylation of the D2/I complex, whereby UBE2T determines mono- versus polyubiquitylation [13], [16].

Apart from FANCL, the many other components of the FA core complex play crucial roles in the assembly and regulation of the complex. In particular FANCM probably targets the FA core complex to sites of DNA damage and, through its own helicase/translocase activity and associated proteins, functions in recognising and remodelling stalled replication forks [17], [18], [19], [20], [21], [22], [23]. FANCM has also been recently implicated in the activation of the ATR/ATRIP kinase-signalling cascade [24], possibly enabling the integration of checkpoint responses with DNA repair at sites of stalled replication.

This paper reports a further level of complexity in the function of individual components of the core FA pathway. We show here that the E2 conjugating enzyme UBE2T and the helicase/translocase FANCM also function in response to UV light-induced DNA damage. Genetic dissections indicate that both genes function with nucleotide excision repair (NER) factors to initiate repair of UV photolesions.

## Results

### UBE2T deficient cells are hypersensitive to a range of DNA damaging agents including UV radiation

**Figure 1:**
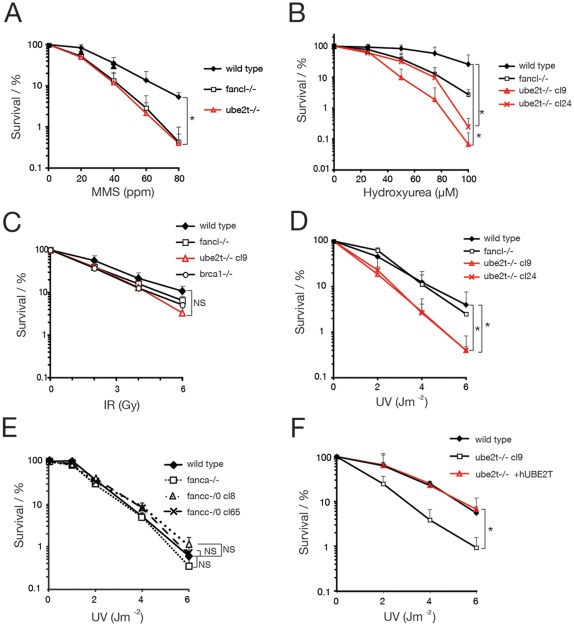
UBE2T deficient cells are sensitive to a range of DNA damaging agents including UV light. (**A**–**F**) Clonogenic survival assays to assess viability of indicated cell lines that have been exposed to MMS (methylmethanesulfonate) (**A**), HU (hydroxyurea) (**B**), IR (ionizing radiation) (**C**) and UV (UV-C, 254 nm) (**D**–**F**). Error bars represent one standard error of the mean from at least three independent experiments. *t*-tests have been performed for indicated survival curves. P≥0.05 not significant (NS), P≤0.05 statistically significant (*), P≤0.01 statistically significant (**);

Our previous work described the generation of a chicken DT40 cell line carrying a disruption of *UBE2T* [15]. This cell line hitherto referred to as ube2t−/− was hypersensitive to the ICL-inducing reagent cisplatin and failed to monoubiquitylate FANCD2. We then tested the viability of ube2t−/− cells following exposure to a battery of different DNA damaging reagents (**Fig.** **1**). These include the replicative stress-inducing reagents methylmethanesulfonate (MMS) and hydroxyurea (HU), ionizing radiation (IR), and UV-C radiation (UV). As anticipated, ube2t−/− cells largely phenocopied sensitivities of a cell line carrying a disruption of the FA core complex gene *FANCL*, fancl−/− (**Fig. 1A–1C**). However, the analyses of two independently generated ube2t−/− cell lines (clone 9 and clone 24) revealed a significantly increased sensitivity to the exposure of UV light whilst this is not the case for fancl−/− cells (**Fig.** **1D**). These results suggest that UBE2T's E2 conjugase activity but not the FA core complex E3 ligase activity is required to protect cells from UV-induced genotoxicity. In agreement with this notion, we did not observe a significant UV sensitivity for cell lines deficient for the FA core components FANCA and FANCC in comparison with isogenic wild type cells (**Fig.** **1E**). A modest UV sensitivity has been recently described for a chicken fancc-/0 cell line [25], however, we could not reproduce this observation with our two independently derived fancc-/0 cell lines (clone 8 and 65). To exclude the possibility that the UV sensitivity of ube2t−/− cells was caused by second site mutations, which might have evolved during the generation of the ube2t−/− cell line, we expressed full length UBE2T cDNA in these cells. As previously shown for cisplatin hypersensitivity [15], cell survival after UV treatment was also fully rescued in ube2t−/− cells expressing UBE2T, suggesting that UBE2T is required to resist UV induced genotoxicity (**Fig.** **1F**).

### UBE2T and FANCM cooperate to protect cells from UV-induced genotoxicity

**Figure 2:**
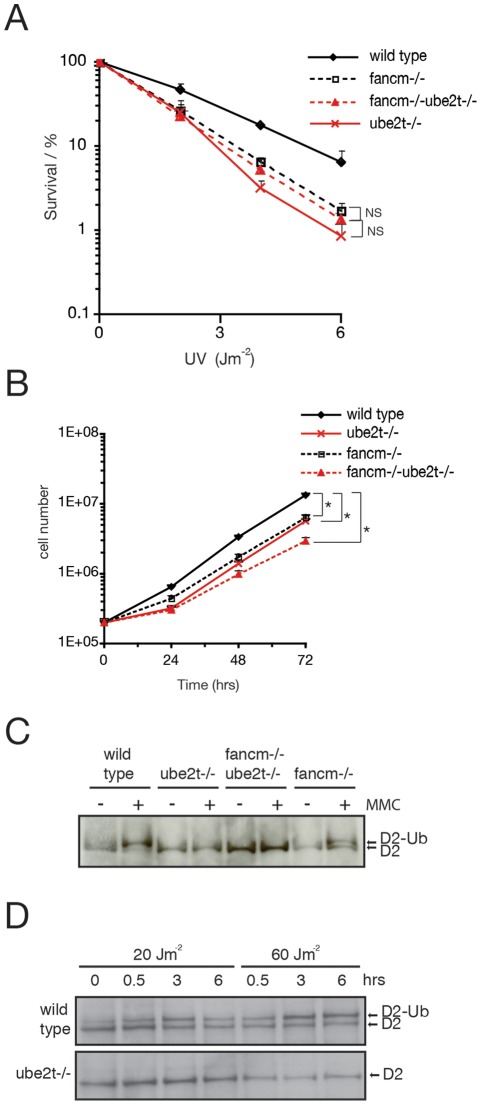
UBE2T and FANCM cooperate to protect cells from UV-induced damage. (**A**) Colony survival of indicated cell lines after exposure to increasing doses of UV light. Error bars represent one standard error of the mean from at least three independent experiments. *t*-tests have been performed for indicated survival curves. P≥0.05 not significant (NS); (**B**) Exponential growth curve of indicated cell lines. Cell density measurements were carried out over a 72 hr time period. Error bars represent one standard error of the mean from at least two independent experiments that were performed in duplicate. *t*-tests have been performed for indicated growth curves with calculated P values P≤0.05 (*); (**C**) Immunoblot analysis to monitor MMC-induced FANCD2 monoubiquitylation. Cells were mock treated (−) or exposed to 150 ng/ml MMC (+) for 18 hrs and whole cell lysates were probed for FANCD2 by immunoblotting. Monoubiquitylated FANCD2 resolves as a slower migrating band (indicated by D2-Ub). (**D**) Immunoblot analysis to monitor UV light-induced FANCD2 monoubiquitylation. Cells were irradiated with indicated doses of UV light and allowed to recover for 0, 0.5, 3 and 6 hrs. Whole cell lysates were probed for FANCD2 by immunoblotting.

We previously reported that a chicken fancm−/− deletion cell line displays sensitivity to UV radiation. FANCM and UBE2T have distinct functions in the FA monoubiquitylation pathway. FANCM is an integral part of the FA E3 ligase complex facilitating the localization of the FA core complex to sites of DNA damage [20], [26], whereas UBE2T interacts with the embedded FANCL component of this E3 ligase complex to monoubiquitylate FANCD2 [10]. In addition, FANCM functions very early in the DNA damage response to ICLs possibly by stimulating the ATR/ATRIP/CHK1 checkpoint responses [24]. A critical question that we set out to resolve is whether the sensitivity to UV light exhibited by ube2t−/− and fancm−/− is because both genes function in a common or distinct response(s) to UV-induced damage. We therefore generated a double knockout cell line that carries disruptions of *UBE2T* and *FANCM* (fancm−/−ube2t−/−) genes. Exposure of this double mutant strain to UV light did not result in increased cellular sensitivity in comparison to either single mutant (**Fig.** **2A**) indicating a genetic link between UBE2T and FANCM in UV damage response. In addition, there is only a minor impact on cell growth in this double mutant (**Fig.** **2B**). As expected the double mutant strain was also not capable of monoubiquitylating FANCD2 in response to MMC and UV-induced FANCD2 monoubiquitylation was abolished in ube2t−/− cells (**Fig. 2C and 2D**). In summary, analyses of the UV sensitivity of the fancm−/−ube2t−/− cells suggest that FANCM and UBE2T cooperate in a common UV damage response pathway.

### UV sensitivity in ube2t−/− cells is not restricted to phases of the cell cycle or due to defective ATR checkpoint activation

**Figure 3:**
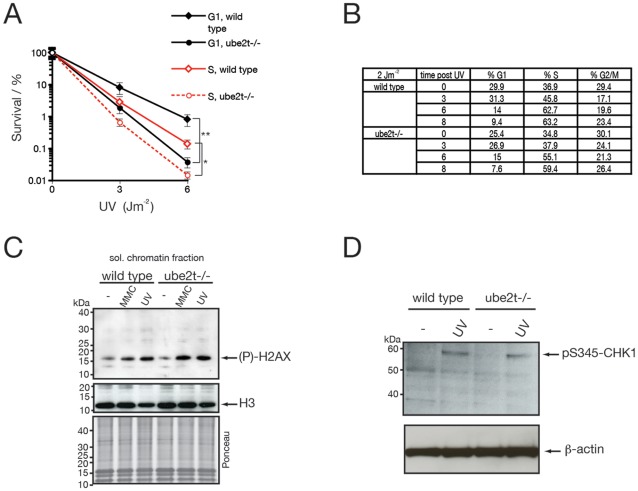
Robust UV-induced checkpoint activation in ube2t −**/**− **cells.** (**A**) Wild type and ube2t−/− cells were arrested in M phase with nocodazole. Cells were then released and synchronized cell populations in either G1 (black) or S (red) cell populations were treated with 0, 3 and 6 Jm^−2^ UV light. Cell survival was determined in a colony survival assay. Error bars represent one standard error of the mean from two independent experiments. In addition *t*-tests have been performed for indicated survival curves. P≤0.05 statistically significant (*) and P≤0.01 statistically significant (**). (**B**) Asynchronous populations of wild type and ube2t−/− cells were irradiated with 2 Jm^−2^, then fixed. Then cells were subjected to Propidium Iodide cell cycle analysis after indicated time points. The estimated percentages of cells in G1, S, G2/M phases are presented in a table. (**C**) Analysis for phospho-H2AX (γ-HA2X) in response to MMC (150 ng/ml) or UV (10 Jm^−2^). Soluble chromatin fractions were prepared from wild type and ube2t−/− cells and subjected to immunoblot analysis. Immunoblot analysis for Histone 3 (H3) and Ponceau S stained nitrocellulose membrane showing equal loading. (**D**) Analysis for phospho-CHK1 (pS345-CHK1) levels in response to UV (10 Jm^−2^). Whole cell lysates were prepared from wild type and ube2t−/− cells and subjected to immunoblot analysis using a phospho-CHK1 (pS345-CHK1) specific antibody. Actin immunoblot analysis was used as a loading control.

Data presented in the previous section places both FANCM and UBE2T in a common pathway in response to UV-induced damage. Recently FANCM was identified as an activator of the ATR/ATRIP/CHK1 checkpoint response following DNA damage during replication [24], [27]. It is therefore possible that UBE2T functions together with FANCM in such a checkpoint response. As the ATR kinase-induced response is largely restricted to the S phase a simple hypothesis would be that UBE2T cells might exhibit differential sensitivity to UV damage when restricted to phases of the cell cycle. To test for a cell phase specific UV sensitivity, DT40 cells were first arrested in M phase with nocodazole, and then following release synchronized cell populations in either G1 or S phase were treated with 0, 3, 6 Jm^−2^ doses of UV light. Cell survival was determined by colony survival assay. The data in **Fig.** **3A** show that UBE2T cells display a sensitivity to UV light irrespective of whether the damage was applied during G1 or S phases. In addition, cell cycle analysis of asynchronous cells exposed to UV light showed that both wild type and ube2t−/− cells exhibited similar dynamics of cell cycle transit. In both instances cells accumulate in the S phase (**Fig.** **3B**). To further confirm that activation of the canonical ATR checkpoint is intact in *UBE2T* knockout cells we tested how efficiently the phosphorylation of H2AX (γ-H2AX) and CHK1 (pSer^345^-CHK1) occurs. Indeed, MMC- or UV-induced γ-H2AX formation and pSer^345^-CHK1 phosphorylation in ube2t−/− cells were intact and indistinguishable from wild type cells (**Fig.** **3C and 3D**). Taken together, these data suggest that UBE2T deficient cells are not defective in checkpoint activation associated with FANCM deficiency.

### The NER gene XPA is epistatic to UBE2T and FANCM in the cellular response to UV-induced genotoxicity

**Figure 4:**
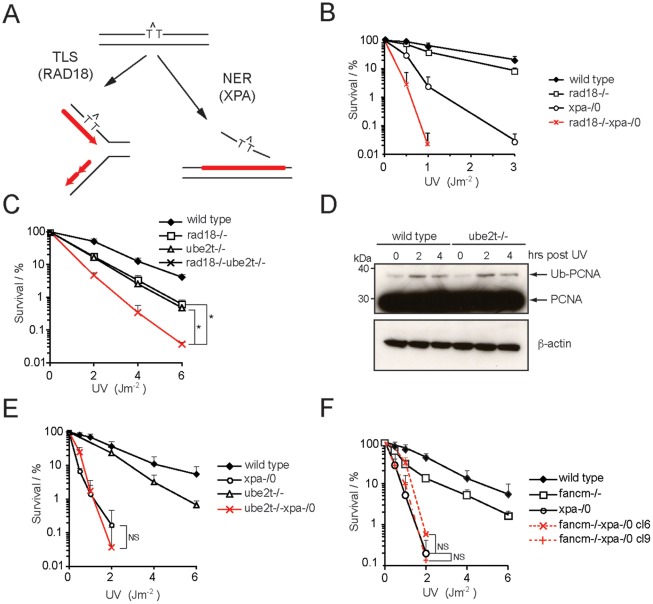
*XPA* is epistatic with *UBE2T* and *FANCM* in response to UV light. (**A**) The two mechanistically different routes to resolve UV photolesions: Translesion synthesis (TLS) and Nucleotide Excision Repair (NER). (**B,** **C**, **E, and F**) Cellular viability after exposure to increasing UV doses was determined for the indicated cell lines by clonogenic survival assays. Error bars represent one standard error of the mean from at least three independent experiments. In addition *t*-tests have been carried out for indicated survival curves. P≥0.05 not significant (NS), P≤0.05 statistically significant (*). (**D**) Immunoblot showing PCNA monoubiquitylation in whole cell lysates of wild type and ube2t−/− cells after exposure to UV (60 Jm^−2^) and the indicated recovery time. Immunoblot for β-actin confirms equal protein loading.

There are two mechanistically different routes to repair (or bypass) UV photolesions in DNA templates – NER and translesion synthesis (TLS) (**Fig.** **4A**). NER excises a short oligomer containing the UV lesion by the coordinated action of the core NER repair factors. The resultant gap is subsequently filled by either Polε or Polδ/κ and then ligated to regenerate the duplex DNA (**Fig.** **4A** **, right**) [28], [29], [30]. TLS bypass is initiated by the RAD6/RAD18 mediated monoubiquitylation of PCNA. Ubiquitylated PCNA then mediates the recruitment of specialized DNA polymerases which then synthesise across the UV induced lesion (**Fig.** **4A** **, left**) [31]. Genetic and biochemical evidence has clearly implicated the FA pathway in facilitating TLS during ICL repair [32], [33]. In addition, the FA pathway operates in close proximity to the replication fork; likewise TLS operates to repair UV damaged DNA following replication fork arrest. We chose a genetic approach to address the key question whether UBE2T and FANCM operate a TLS or NER route to resist UV photolesions. Deletions of either the TLS regulating gene *RAD18* or the NER gene *XPA* in DT40 cells cause UV sensitivity **(** **Fig.** **4B** **)** [34], [35], [36]. Importantly, the rad18−/−xpa-/0 double mutant is more sensitive than the corresponding single mutant cell lines, confirming the notion that RAD18 and XPA act in parallel pathways. We next generated double mutants by combining disruptions of *UBE2T* with either *RAD18* or with *XPA*. Both of these double mutant strains, rad18−/−ube2t−/− and ube2t−/−xpa-/0, were viable. We then tested the double and corresponding single mutant cell lines for their sensitivity to UV light exposure. Given the genetic link between FA and TLS in response to ICLs, we were surprised to find that the combined deletion of *UBE2T* and *RAD18* (rad18−/−ube2t−/−) was additive with respect to individual mutants after UV-induced damage (**Figure 4C**). It is also noteworthy that UBE2T does not contribute to UV-induced PCNA monoubiquitination, a key modification that promotes TLS **(** **Fig.** **4D** **)**. In complete contrast, the analysis of ube2t−/−xpa-/0 cells showed that *UBE2T* is epistatic with *XPA* in response to UV light (**Fig.** **4E**). To complete this analysis we then generated a fancm−/−xpa-/0 double mutant (two independently derived cell clones 6 and 9), which showed that *FANCM* is epistatic with *XPA* as well (**Fig.** **4F**). Hence we concluded from these genetic analyses that both *UBE2T* and *FANCM* function not in the TLS but in the NER response to UV damage in chicken DT40 cells.

### UBE2T deficiency affects the NER-mediated removal of cyclobutane pyrimidine dimers

**Figure 5:**
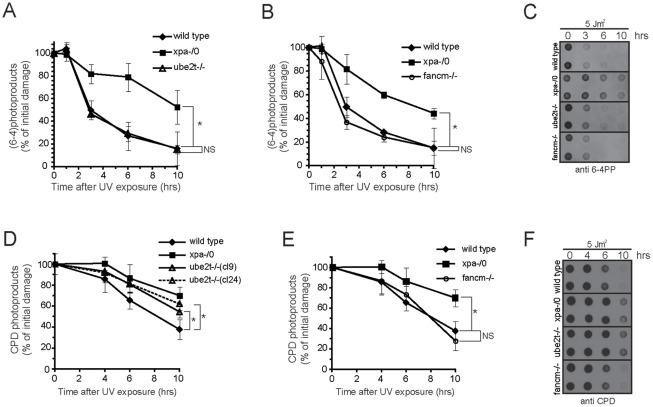
UBE2T deficiency affects NER-mediated removal of cyclobutane pyrimidine dimers (**CPDs**)**.** Genomic DNA was isolated from UV irradiated (5 Jm^−2^) cell lines after indicated time points and analysed for the presence of photoproducts as described in Material and Methods. (**A**)(**B**) show the progression of (6–4)PP photoproduct removal and (**D**)(**E**) the removal CPDs. The amounts of both types of lesions are presented as percentages of those at time 0. (**C**) and (**F**) show representative immunoblots for (6–4)PPs and CPDs respectively. Error bars represent standard error of the mean from three (for (6–4)PP) and six (for CPDs) independent experiments. *t*-tests have been performed for indicated curves. P≥0.05 not significant (NS), P≤0.05 statistically significant (*).

Genetic evidence places UBE2T and FANCM functionally in the NER mediated UV damage response pathway. We would therefore predict that the persistence of UV photolesions is responsible for the genotoxicity observed in ube2t−/− and fancm−/− cells. NER is known to eliminate a wide variety of helix-distorting DNA base lesions. The two predominant UV photolesions, cyclobutane pyrimidine dimers (CPD) and pyrimidine-pyrimidone (6–4) photoproducts (6–4)PP, occur when covalent linkages are formed between adjacent pyrimidines [37]. CPDs cause only minor duplex destabilisation and render CPDs the most difficult photolesion to detect. Hence, these lesions are highly mutagenic and the major factor of skin carcinogenesis [38]. The presence of these photoproducts can be detected and monitored in genomic DNA with specific antibodies directed against them [39]. We isolated genomic DNA from untreated (0 Jm^−2^) and UV treated (5 Jm^−2^) cells after the indicated time points. We then dot-blotted the genomic DNA onto nitrocellulose membrane, followed by an immunoblot analysis with anti-CPD or anti-(6–4)PP antibodies and quantification of the autoradiogram (**Fig.** **5C and 5F**). We note here that the chicken genome, in contrast to mammalian ones, encodes a potential photolyase homologue. Photolyases reverse the photoproduct formation in a visual light dependent manner. Precaution was therefore taken to minimize exposure to visual light during the experimental procedure. In particular cells were kept in the dark during recovery time. As shown in **Figure** **5**, ∼60% of (6–4)PP photolesions and CPDs were removed efficiently in wild type cells within 3 and 10 hrs respectively. In contrast, the removal was clearly delayed in xpa-/0 cells and on average 60–70% of photolesions persisted. The removal of (6–4)PP photolesions in genomic DNA from ube2t−/− and fancm−/− cells was indistinguishable from the wild type cells. We concluded that these cell lines are unimpaired in the excision of (6–4)PP photolesions (**Fig.** **5A and 5B**). In contrast the elimination of CPD photolesions in the two independent clones of ube2t−/− cells (cl9 and cl24) was impaired (**Fig.** **5D**). This defect was most prominent 10 hrs after UV exposure. We measured 35.1±12.9% of remaining CPDs in wild type cells, whereas ube2t−/− (cl9) and (cl24) still have 54.4±1.5% and 62.0±9.5% unrepaired CPDs, respectively. Interestingly, and rather to our surprise given the epitasis between UBE2T and FANCM, there was no detectable defect in CPD photolesion removal in the fancm−/− strain (**Fig.** **5E**). In summary these results suggest a distinct function for UBE2T and FANCM in the NER pathway. UBE2T most likely has a regulatory function at or upstream of the CPD excision step, whereas FANCM may act downstream of the excision step.

### UBE2T is not required for UV-induced XPC polyubiquitylation

**Figure 6:**
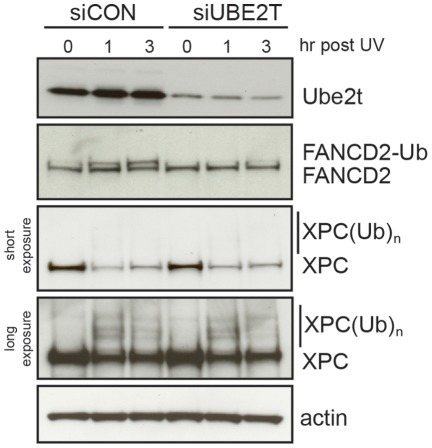
UBE2T is not required for UV-induced XPC polyubiquitylation. Immunoblot analysis for UV light induced XPC ubiquitylation in siUBE2T and non-target siCON transfected U2OS cells. Lysates of UV (20 Jm^−2^) irradiated cells were prepared after indicated chase times. Immunoblot for UBE2T and FANCD2 shows efficient knock down of UBE2T protein levels and defective UV-induced FANCD2 monoubiquitylation. Two time exposures are presented for anti-XPC.

Our analysis of ube2t−/− cells uncovered a NER specific defect in processing CPD photolesions. This particular CPD repair defect accompanied by a mild UV hypersensitivity has also been described for cells derived from Xeroderma Pigmentosum E (XP-E) patients (reviewed in [40]). XP-E cells lack a functional DDB2 protein, which is part of the DNA binding UV-DDB complex. The UV-DDB complex enhances binding of the XPC/HR23 complex to CPD photolesions in order to promote the formation of the NER pre-incision complex. More recently, DDB2 was co-precipitated as an adapter of the DDB1-Cul4-Roc1 complex, a member of the cullin E3 RING ligases [41]. This E3 ubiquitin ligase defines an ubiquitin-signalling pathway that promotes polyubiquitlation of XPC, which enhances its affinity to damaged DNA [42]. The identity of the physiological relevant E2 involved in this ubiquitylation step is ambiguous and we therefore wanted to test if UBE2T forms a functional E2/E3 module with DDB1^DDB2^-Cul4A-Roc1. We could technically not monitor UV-induced polyubiquitylation of XPC in DT40 cells. We therefore knocked down UBE2T by siRNA in U2OS cells. As shown in **Figure** **6**, knock down of UBE2T protein was sufficient to lead to impaired UV-induced FANCD2 ubiquitylation. High molecular weight species of XPC, which were indicative of ubiquitylated XPC, could be readily detected 1 hr after UV damage. However, siUBE2T depletion neither altered the timing nor the abundance of XPC polyubiquitylation. These data suggest that UBE2T plays no role in DDB1^DDB2^-Cul4A-Roc1 mediated XPC ubiquitylation. Therefore UBE2T most likely functions in a potentially new ubiquitin-signalling pathway converging on NER.

## Discussion

The comprehensive genetic dissection analysis in avian DT40 cells presented here reveals that two intrinsic components of the FA repair pathway, UBE2T and FANCM, also function with NER factors to repair UV induced DNA damage. In particular we have identified that UBE2T deficiency impinges on the efficient repair of one type of UV photolesion – CPD. To our knowledge this is the first demonstration that FA genes function in NER. We propose that UBE2T may constitute a distinct DNA damage induced ubiquitin-signalling pathway that converges on an early step in NER. An important question is whether we may extrapolate our findings to mammalian cell. There are obvious limitations of the DT40 system, which have to be taken into account when interpreting results in this context. Firstly, DT40 is a lymphoblastoid cell line and the conservation of pathways and subtle mechanistic factors may alter the repair pathway choice. Secondly, it is a transformed cell line that lacks p53, which can have a significant impact on the DNA damage response. The question then arises as to whether there is any evidence that UBE2T and FANCM are functionally linked to NER in human cells? A human lymphoplastoid cell line (EUFA867) deficient in FANCM has been shown to be UV sensitive to the same degree as a XP-C cell line (XP3BE), suggesting that human FANCM is required to protect cells from UV-induced damage [43]. Further studies will be needed to address a role of human FANCM in NER. In contrast, no patient with defective UBE2T has been identified yet and no UBE2T mouse knock out cell lines have been described either. At this stage we can only speculate about a function of UBE2T in the human NER pathway. However, our results derived from the DT40 system will form the foundation for further studies of mammalian UBE2T and FANCM in NER.

Both FANCM and UBE2T, albeit to varying levels, regulate the ICL-induced damage response by monoubiquitylating FANCD2 [10], [15], [20], [26]. Inactivation of FANCM in a variety of organisms compromises the FA core complex integrity, which severely reduces the efficiency of FANCD2 monoubiquitylation. It is still unclear whether the consequences of FANCM deficiency on FANCD2 monoubiquitylation is solely due to a compromised core complex function or to defective activation of the ATR/ATRIP checkpoint pathway or indeed due to both [24]. FANCM is one of the most conserved proteins in the FA pathway [44] [26], [45] and is clearly involved in other DNA repair pathways, such as in the suppression of crossover recombination (e.g. sister chromatid exchanges), and also resistance to DNA double strand break-inducing agent camptothecin (CPT) [46], [47]. Our previous work implicates the helicase domain of FANCM as being critical for these functions. How then might FANCM contribute to NER? The observation that FANCM is able to translocate along DNA and also bind and remodel branched DNA structures prompts us to speculate that it may function at an early step in NER such as in damage recognition or chromatin remodelling of UV damaged DNA. However, the efficient removal of photolesions in the absence of FANCM suggests a function downstream of lesion recognition and elimination. Nevertheless it is clear from our genetic analysis that FANCM functions in the same branch of NER regulation as UBE2T (**Fig.** **2**). The role of UBE2T in the FA pathway is much better understood since it is the critical E2 conjugating enzyme catalyzing FANCD2 monoubiquitination. Loss of UBE2T completely abolishes the FA ubiquitylation response [10], [15], and importantly results in failure to incise DNA at sites adjacent the ICLs [33]. Therefore, in a way both FANCM and UBE2T eventually regulate the assembly/disassembly or the function of a DNA incision nuclease complex.

The well-studied function of both UBE2T and FANCM in the FA pathway is in stark contrast to their new roles in NER. Our studies do however allow us to narrow down the point at which at least UBE2T is likely to function in NER. The specific defect we observe in the reduced efficiency of the removal of CPD photolesions suggests that UBE2T must at some point facilitate the excision of this lesion from DNA. We were struck by the parallels between these observations and the now well-understood role of the XPE gene product – which, when mutated in humans, leads to a mild version of Xeroderma Pigmentosum [40]. In this instance XPE is in a molecular complex with the DDB1-Cullin4A-ROC1 multi-subunit E3 ubiquitin RING ligase [41], [48]. The main role of this E3 ligase complex in NER appears to be in the polyubiquitylation of the XPC protein in response to UV damage [42]. Polyubiquitinated XPC is not targeted for proteasomal degradation instead it appears that ubiquitylation enhances XPC's affinity to UV-damaged DNA templates and therefore its capacity to initiate NER. It is striking that XPE mutant cells are particularly defective at the removal of CPD photolesions – just like we observe for UBE2T [40]. It is thought that this response is needed to deal with lesions such as CPDs, which are not as helix distorting as 6–4PP products. However, it is clear from our analysis that UBE2T does not regulate XPC polyubiquitylation (**Fig.** **6**). The DDB1-Cullin4A-ROC1 E3 ligase can also form with the substrate receptor Cockayne syndrome A (CSA) protein an alternative complex, DDB1^CSA^-Cullin4A-ROC1, which serves in transcription-coupled NER (TC-NER) [41]. TC-NER is an alternative pathway of NER that is involved in the repair of actively transcribed DNA strands. After exposure to UV light, DDB1^CSA^-Cullin4A-ROC1 accumulates at sites of stalled RNA polymerase II to promote DNA repair and subsequent RNA synthesis recovery [49]–[50]. A possible explanation of the modest impairment of NER, which we observe in ube2t−/− cells, would be a specific function of UBE2T with DDB1^CSA^-Cullin4A-ROC1 in TC-NER. This would also be in agreement with a UV sensitivity of ube2t−/− cells in G1 phase (**Fig.** **3A**). However, this possibility is unlikely as we could show that UBE2T knock down in U2OS cells did not affect RNA synthesis recovery after UV irradiation (**Fig.** **S1**). Cumulatively, we have no evidence that UBE2T is functionally linked with DDB1-Cullin4A-ROC1 ligase complexes and therefore we suggest a new UBE2T-dependent ubiquitin-signalling pathway in response to UV photolesions.

The mechanistic concept of ubiquitin transfer onto target proteins depends on the assembly of a functional enzyme complex between an E2 conjugating enzyme and an E3 ligase. A key question, for future studies, will be therefore to address which E3 ligase cooperates with UBE2T to promote NER. In addition it will be crucial to identify the substrates of such an UBE2T/E3 ligase complex since this will uncover the molecular nature of how ubiquitylation contributes to NER regulation. It is intriguing that the two components of the FA pathway, that are ultimately linked to incision complexes for DNA crosslink repair, should also contribute in such a defined manner to NER.

## Materials and Methods

### DT40 cell culture

DT40 cells were cultured at 37°C, 5% CO_2_ in RPMI 1644 supplemented with 7% fetal calf serum, 3% chicken serum (GIBCO), 10 µM β-mercaptoethanol and in the presence of penicillin/streptomycin at 37°C, 5% CO_2_.

### Generation of fancm−/−ube2t−/−, rad18−/−ube2t−/−, ube2t−/−xpa-/0, and fancm−/−xpa-/0 cells

All cell lines described here are derivatives of the chicken B cell line DT40 [51]. fancm−/− cells [26] were sequentially transfected with *UBE2T-his* and *UBE2T-bsr* targeting constructs [15] to obtain a fancm−/−ube2t−/− double deletion cell line. To disrupt the one allele of the XPA gene (on chromosome Z) in the fancm−/− or ube2t−/− deficient cell lines we used the *XPA-puro* targeting construct (kindly provided by the lab of Shunichi Takeda)[34]. To generate the rad18−/−ube2t−/− double deletion strain, *UBE2T-puro* and *UBE2T-bsr* targeting constructs were sequentially transfected into the rad18−/− genetic background (rad18−/− cell line kindly provided by Minoru Takata) [34]. *fancc-/0* cells have been described in [32]. The rad18−/−xpa-/0 cell line was kindly provided by Julian Sale [52]. Transfections, selections, and Southern blot analyses of locus-targeted DT40 clones were carried out as previously described [32].

### Colony survival assay, cell growth determination and flow cytometry

Colony survival assays for cisplatin, mitomycin C and IR (ionizing radiation) sensitivities were performed as previously described [32]. Sensitivities to UV-light exposure were determined as previously described [46]. Flow cytometry, cell growth and doubling time measurements were carried out as previously described [32].

### siRNA, immunoblot analyses and antibodies

Where indicated, whole cell lysates were prepared by lysis of cells in RIPA buffer (phosphate buffered saline (PBS), 1% Nonidet-P40, 0.1% sodium dodecyl sulfate, 0.5% sodium deoxycholate and protease inhibitor cocktail, (ROCHE)), followed by centrifugation. Where necessary, 5 mM N-ethylmaleimide was also included to inhibit deubiquitinase activity during lysis. Clarified lysates were separated on 4–12% Bis-Tris-PAGE (Invitrogen) gels or, in the case for FANCD2 detection, resolved on 3–8% Tris-Acetate-PAGE gels (Invitrogen). Preparations of soluble chromatin fractions from DT40 cells were carried out as described previously [15]. siRNA knock down of UBE2T was carried out as described in [10]. The following primary antibodies for immunoblot analyses were used in this study: FANCD2 [32], pS345Chk1 (Cell Signalling Technology), γ-H2AX (Bethyl), XPC and PCNA (Abcam), and β-actin (Cell Signalling Technology), (6–4)photoproducts (Cosmo Bio Co.), cyclobutane pyrimidine dimers (CPDs) (Sigma). To generate antisera specific to human UBE2T, sheep were immunized with purified recombinant GST-UBE2T. Antisera were pre-cleared on a GST-coupled Affigel-15 matrix (Biorad) and then affinity purified on an Affigel-15 matrix coupled to UBE2T.

### Measurement of CPD and (6–4) photoproducts

The NER capacity in DT40 cells was determined by estimating the amount of CPD and (6–4) photoproducts in UV-irradiated cells.5×10^6^ cells in PBS were UV-irradiated (5 Jm^−2^) and then incubated for indicated time points (0, 1, 3, 4, 6 and 10 hrs) in complete medium at 37°C in the dark to allow repair to take place. Genomic DNA (gDNA) was extracted using the PUREGENE DNA Purification Kit (Gentra Systems) following the manufacturers protocol. It should be noted that gDNA samples also contain DNA from resumed DNA replication after UV-irradiation and thus will dilute damaged DNA at later time points and will contribute to a repair-independent reduction of photolesion measurements. gDNA samples were diluted to 1 µg/µl in DNA Hydration Solution (Gentra Systems), boiled for 10 minutes and chilled on ice. 3 µl of the denatured gDNA were dot-blotted onto Hybond^+^ (GE Healthcare) membrane. DNA was fixed to the membrane for 1 hr at 80°C in a hybridization oven. For subsequent immunodetection of the photoproducts, the membrane was blocked in 1x Tris buffered saline (TBS) (supplemented with 0.1% Tween-20, 0.75% milk powder) for 1 hr at RT, and then hybridized with either anti-CPD or anti-(6–4) PP antibodies (1 µg/ml in 1x TBS, 0.02% Tween-20) at ambient temperature for 1 hr. The membrane was then washed 3x with 1x TBS (0.05% Tween-20). The primary antibody was detected using horseradish-peroxidase-conjugated anti-mouse antibody. Autoradiograms for different time exposures were taken to obtain non-saturated signals, scanned (CanonScan 8800F, Canon) and quantified using the ImageJ software.

### Measuring recovery of RNA synthesis by immunofluorescence

U2OS cells were grown on 13 mm diameter glass coverslips in 6 cm^2^ dishes before being treated with non-targeting siRNA or siRNA against UBE2T or XPA (Dharmacon SMARTpool). Recovery of RNA synthesis was measured according to the protocol for siRNA screening described recently [53]. Briefly, after 48 h treatment with siRNA, cells were left untreated or treated with 5 J/m^2^ UV and allowed to recover for 4 h in media containing 1% fetal bovine serum (FBS) to allow RNA synthesis to recover. Cells were then incubated in serum free media containing 100 µM 5-ethynluridine (EU) (Invitrogen) to label newly synthesized RNA for 2 h. Cells were fixed and permeabilized by incubating in PBS containing 2 % paraformaldehyde, 0.5 % Triton X-100 and 300 mM sucrose for 20 minutes on ice. Cells were blocked with 10 % FBS in PBS for 30 minutes before incubation for 1 hour with 25 µM of the Alexa Fluor 488 Azide fluorophore (Invitrogen). After extensive washing in PBS with 0.05% Tween-20, coverslips were mounted on glass slides and stained with DAPI-Hydromount (Vectashield). Cells were then viewed on a Deltavision DV3 wide-field deconvolution microscope. EU fluorescence was measured using ImageJ software.

## Supporting Information

Figure S1 **UBE2T depletion does not cause defects in recovery of RNA synthesis following UV irradiation.** U2OS cells grown on glass coverslips were depleted of UBE2T or XPA by siRNA and the recovery of RNA synthesis was measured after irradiation with 5 J/m^2^ UV. Newly synthesized RNA was labeled with 5-ethynluridine (EU) that was covalently coupled to a fluorophore for detection by immunofluorescence. (**A**) Representative images of EU fluorescence in cells treated with the indicated siRNA and exposed to UV or not (untreated). (**B**) Quantification of the data represented in (**A**). EU fluorescence of at least 100 nuclei was measured using ImageJ software and the percentage recovery of RNA synthesis for each siRNA treatment is shown. Error bars represent one standard error of the mean from two independent experiments. Statistical significance was calculated using a *t*-test. Not statistic significant (NS), statistic significant P≤0.01 (**).
(TIF)
