# Supplementary figures and images for: The Fanconi Anaemia Components UBE2T and FANCM Are Functionally Linked to Nucleotide Excision Repair
Source: PLoS One. 2012 May 15;7(5):e36970. doi: 10.1371/journal.pone.0036970 (PMC3352854; doi:10.1371/journal.pone.0036970)

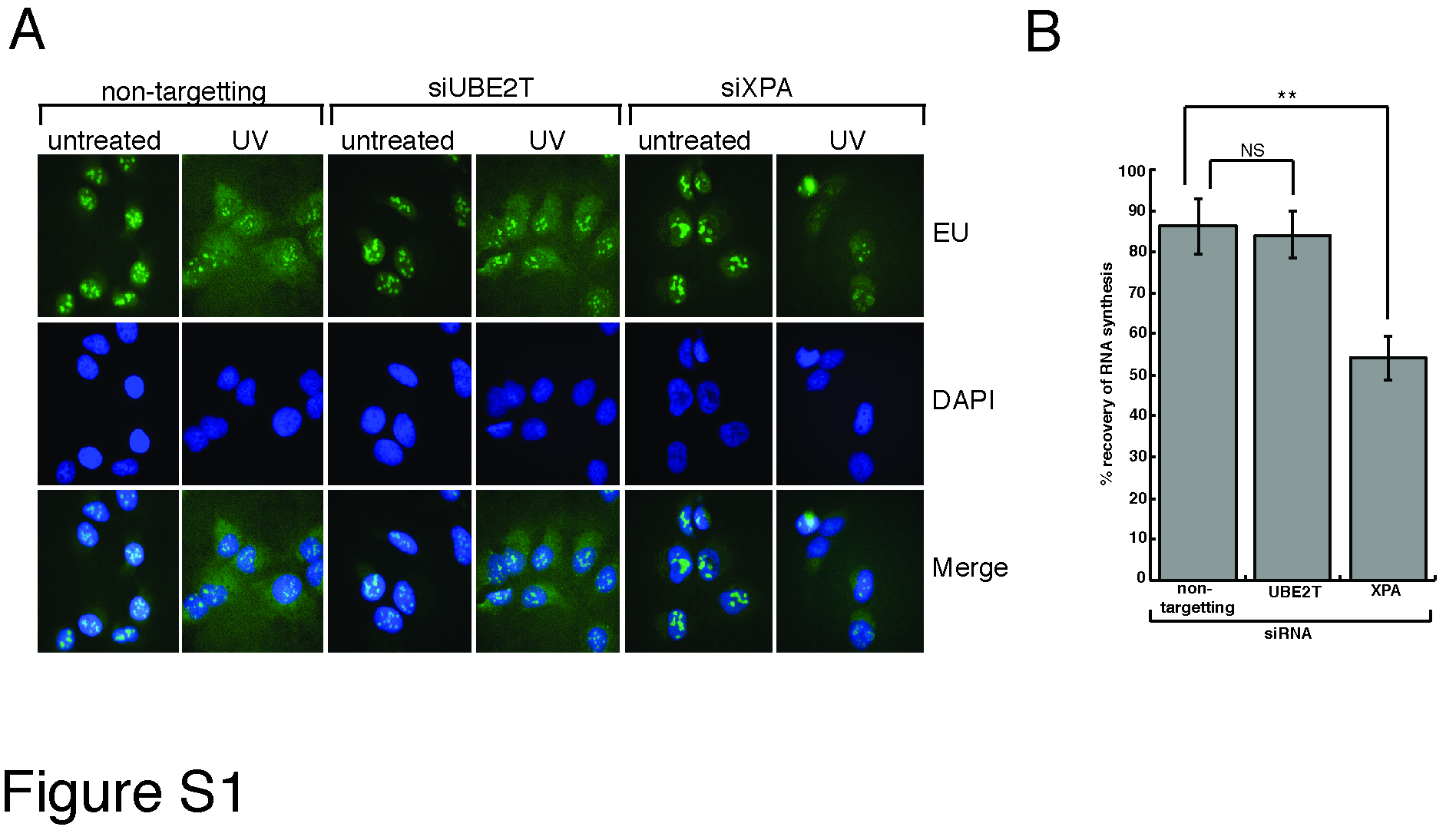

Supplement: Figure S1 — UBE2T depletion does not cause defects in recovery of RNA synthesis following UV irradiation. U2OS cells grown on glass coverslips were depleted of UBE2T or XPA by siRNA and the recovery of RNA synthesis was measured after irradiation with 5 J/m2 UV. Newly synthesized RNA was labeled with 5-ethynluridine (EU) that was covalently coupled to a fluorophore for detection by immunofluorescence. (A) Representative images of EU fluorescence in cells treated with the indicated siRNA and exposed to UV or not (untreated). (B) Quantification of the data represented in (A). EU fluorescence of at least 100 nuclei was measured using ImageJ software and the percentage recovery of RNA synthesis for each siRNA treatment is shown. Error bars represent one standard error of the mean from two independent experiments. Statistical significance was calculated using a t-test. Not statistic significant (NS), statistic significant P≤0.01 (**). (TIF) [file pone.0036970.s001.tif]
